# A Deep Probabilistic Sensing and Learning Model for Brain Tumor Classification With Fusion-Net and HFCMIK Segmentation

**DOI:** 10.1109/OJEMB.2022.3217186

**Published:** 2022-10-25

**Authors:** M. V. S. Ramprasad, Md. Zia Ur Rahman, Masreshaw Demelash Bayleyegn

**Affiliations:** Koneru Lakshmaiah Education FoundationK L University 207673 Guntur 522302 India; GITAM (Deemed to be University) 28668 Visakhapatnam AP 522502 India; Department of Electronics and Communication Engineering, Koneru Lakshmaiah Education FoundationK L University 207673 Vaddeswaram Guntur 522502 India; Center of Biomedical Engineering, Addis Ababa Institute of TechnologyAddis Ababa University 37602 Ethiopia

**Keywords:** Brain tumor segmentation, classification, feature extraction, deep learning convolutional neural network, robust edge analysis

## Abstract

*Goal:* Implementation of an artificial intelli gence-based medical diagnosis tool for brain tumor classification, which is called the BTFSC-Net. *Methods:* Medical images are preprocessed using a hybrid probabilistic wiener filter (HPWF) The deep learning convolutional neural network (DLCNN) was utilized to fuse MRI and CT images with robust edge analysis (REA) properties, which are used to identify the slopes and edges of source images. Then, hybrid fuzzy c-means integrated k-means (HFCMIK) clustering is used to segment the disease affected region from the fused image. Further, hybrid features such as texture, colour, and low-level features are extracted from the fused image by using gray-level cooccurrence matrix (GLCM), redundant discrete wavelet transform (RDWT) descriptors. Finally, a deep learning based probabilistic neural network (DLPNN) is used to classify malignant and benign tumors. The BTFSC-Net attained 99.21% of segmentation accuracy and 99.46% of classification accuracy. *Conclusions:* The simulations showed that BTFSC-Net outperformed as compared to existing methods.

## I. Introduction

World Health Organization [1], the National Brain Tumor Society [2], and the Indian Society of Neuro-Oncology [3] classified that brain tumor as one of the deadliest as it is an uncontrollable growth of a malignant brain cell. Brain tumor incidence has risen sharply in the last 30 years, impacting millions of deaths globally.

For example, 241037 deaths occurred in 2020 [4]. Thus, early diagnosis increases treatment options and survival chances [5]. Tumors may be treated with radiation, surgery, chemotherapy, or a combination of clinical methods [6]. Brain tumors are scanned to identify them, and the most frequently utilized brain imaging methods [7] are MRI and CT. Identifying a tumor from normal brain tissue is crucial and the ability to derive lesion properties from normal tissues helps diagnosis [8]. Lesion size, texture, shape, and placement vary based on person to person. To provide a diagnosis, images must be classified and categorized with tissue segmentation in manual, semi-automated, and completely automated forms. A radiologist manually segments it [9] and expert segmentation needs several enhanced datasets and pixel profiles to identify the damage border. Completely automated machine learning methods are preferable. So, naive bayes, decision, tree, random forest, and support vector machines (SVM) were used as glioma supervised classifiers. Traditional machine learning fails to segment and categorize.

Multimodal image fusion [10] and pixel-to-pixel fusion happen in the spatial domain. There is also a nonsubsampled shearlet transform domain (NSST) and a maximum and minimum for the fusion process by using weighted pixels [11]. Pixel action sets the weights in order to choose the most active pixels, where local laplacian energy and phase congruency are utilized [12]. Further, the original images are squared by utilizing a parameter-adaptive pulse-coupled neural network with average weighting [13]. This image fusion method uses maximum average mutual information. Several works [14] present morphological component analysis-based convolutional sparsity (MCA-CS) for image fusion, in which the first MCA-CS component of each input image is multiplied independently and color, information, and brightness extraction may occur. Several methods are used to do the preprocessing operation. These include laws of texture energy measures (LTEM) [15], graph filter and sparse representation (GFSR) [16], dual-branch CNN (DB-CNN) [17], Gabor filtering based separable dictionary learning (GF-SDL) [18], and local difference in non-subsampled domain LDNSD [19], [20].

Wavelets operate poorly near edges and texturing areas where there is no phase information in the segmentation operation. Further, improved U-Net [21] employed considerable VGG-16 based data augmentation to improve segmentation accuracy. A Dice-based loss function to predict output label using both local and contextual information extracted by using the triple intersecting U-Nets (TIU-Net) architecture in [22]. Cross-modality deep feature learning (CMDFL) [23] with a bat-optimized loss function was used to improve how well the segmentation worked. In addition, an efficient 3D residual neural network (ERV-Net) [24] and a multiencoder network (MENet) [25] were developed for better segmentation with low computational cost. Deep multi-task learning (DMTL) [26] with tiny kernels was also proposed to be done with the CNN encoder-decoder. Due to the small training dataset, MENet and DMTL used the autoencoder branch to guide and regularize the encoder component. In addition, a latent correlation representation learning (LCRL) based variational autoencoder with endpoint clustering is proposed in [27]. A few more segmentation approaches are fast level set-based CNN (FLS-CNN) [28], Bayesian fuzzy clustering with hybrid deep autoencoder (BFC-HDA) [29], and symmetric-driven adversarial network (SDAN) [30]. But the SDAN method suffers from reduced accuracy.

Further, multiscale CNN (MS-CNN) [31] and deep learning with synthetic data augmentation (DLSDA) [32] are proposed for brain tumor classification. But, the accuracy of tumor detection must be enhanced and classified to be identified. Moreover, fine-tuning-based transfer learning (FTTL) [33] is used to differentiate meningioma from non-meningioma brain images with a dice coefficient index [34]. The transfer learning CNN (TL-CNN) [35] model was used to classify brain tumors into three types. The tumor was expanded, then ring-divided and T1-weighted contrast-enhanced MRI was used to modify an existing pre-trained network [36]. This approach can automatically classify glioma brain tumors. The deep-CNN (DD-CNN) [37] model was developed for identifying brain tumors in MRI images. Here, 3D-CNN [38] was developed using MRI images of meningioma and pituitary tumors based on CNN-multi-scale analysis. Furthermore, generative adversarial networks based on variational autoencoders (GAN-VE) [39] and hybrid deep neural networks (HDNN) [40] for improved performance of the classification process. These traditional methods still need to be made better, which is why they don't work well enough for segmenting and classifying.

*Problem statement:* As the size of the dataset grows larger, conventional models suffer from high computational complexity. Further, the fusion, segmentation, and classification performance of conventional approaches needs to be enhanced. The novelty is that there is no common method for fusion, segmentation, feature extraction, and classification as of now. Furthermore, in the literature, these combinations of hybrid methods are not published. To overcome these problems, the novel contribution of the article is as follows:

• A novel BTFSC-Net model is developed with the preprocessing, fusion, segmentation, and classification stages, which is a new design and has not been developed by other authors yet.
• Initially, HPWF was developed for the removal of various noises from MRI and CT medical images, which also enhanced the contrast, brightness, and color properties.
• Then, a DLCNN-based fusion network is used to fuse the preprocessed MRI and CT scans with the REA analysis, which improves the region of tumor.
• In addition, HFCMIK is used to segment the tumor region from the fused outcome, so an accurate area of brain tumor is detected.
• Finally, DLPNN is used to classify the benign and malignant tumors from the GLCM and RDWT trained features.
• The results of the simulation show that the suggested strategy worked better than the other options.

The rest of the manuscript is organized as follows: Section II deals with the proposed BTFSC-Net analysis. Section III deals with results and discussions, along with performance comparisons. Section IV discusses the conclusion and future research.

## II. Materials and Methods

**Fig. 1.**
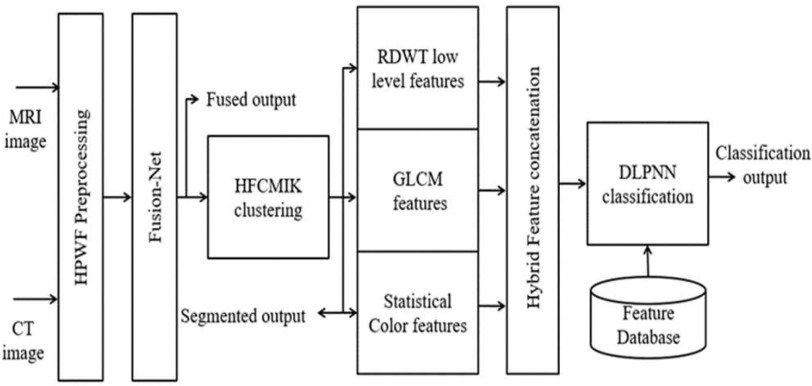
Proposed brain tumor fusion with segmentation and classification model.

This section gives a detailed analysis of brain tumor fusion with segmentation and classification methods. Fig. 1 shows the workflow of the proposed BTFSC-Net methodology, and Table I presents the proposed algorithm. Prior to any further processing, medical images are subjected to a HPWF, which is used to eliminate noise from medical images. The DLCNN-based fusion network is being developed for the fusion of MRI and CT scans with maintaining REA capabilities. Here, REA is utilized here because it is necessary to detect the slopes and borders of the brain images in this case. In order to segregate the illness affected area from the fused image, HFCMIK clustering is applied. Furthermore, hybrid features are derived by combining GLCM, RDWT based statistical color features from the fused image. A DLPNN is utilized to categorize benign and malignant tumors.

**TABLE I:** Proposed Brain Tumor Fusion With Segmentation and Classification Algorithm

**Input:** MRI and CT brain images
**Output:** Classified outcome
**Intermediate outcomes:** Fused and Segmented outcomes
**Objective Evaluation-Set 1:** PSNR, UQI, MSSSIM, RMSE, VIF, SSIM, MSE.
**Objective Evaluation-Set 2:** SPE, SEN, SACC, SDIC, SJAC, SMCC of segmentation
**Objective Evaluation-Set 3:** CACC, CAUC, CSEN, CSPE, SPR of classification.
**Step 1:** Perform a preprocessing operation using HPWF for the removal of various noises from MRI and CT medical images, which also enhances the contrast, brightness, and color properties.
**Step 2:** Fusion-Net is used to fuse the preprocessed MRI, and CT images with the REA analysis, which improves the region of tumor.
**Step 3:** In addition, HFCMIK is used to segment the tumor region from the fused outcome, so an accurate area of brain tumor is detected. **Step 4:** Finally, DLPNN is used to classify the benign and malignant tumors from the GLCM, and RDWT trained features.
**Step 5:** Perform the objective and subjective evaluation.

### A. Hybrid Probabilistic Wiener Filter-Based Pre-Processing

The HPWF successfully enhances the image by removing noise from images using Gaussian mask kernels. Table 2 shows the HPWF algorithm. Images are made up of pixels.

**TABLE II:** Hybrid Probabilistic Wiener Filter Algorithm

**Input:** Noisy image ${X}_{ij}$
**Output:** Denoised image ${Y}_{ij}$
**Step 1:** Consider image ${X}_{ij}$ with random noise properties.
**Step 2:** Estimate ${\sigma }_{GN}$ by convolution with mask as follows: \begin{equation*} {\sigma }_{GN} = \frac{1}{{MN}}\mathop \sum \limits_{i = 1}^M \mathop \sum \limits_{j = 1}^N {\left| {X*MASK} \right|}_{i,j} \tag{2} \end{equation*}
**Step 3:** Choose the mask size based on standard noise threshold as follows: \begin{equation*} w = \left\{ {\begin{array}{c} {3 \times 3,{\sigma }_{GN} < 20}\\ {5 \times 5,{\sigma }_{GN} \geq 20} \end{array}} \right. \tag{3} \end{equation*}
**Step 4:** Apply the mean filtering operation using local mask (R). \begin{equation*} {\tilde{X}}_{i,j} = \frac{1}{R}\mathop \sum \limits_{i = 1}^R \mathop \sum \limits_{j = 1}^R {X}_{i,j}*{w}_{i,j} \tag{4} \end{equation*}
**Step 5:** Estimate the differenced image by performing sliding window subtraction operation. \begin{equation*} {D}_{i,j} = \left| {{X}_{i,j} - {{\tilde{X}}}_{i,j}} \right| \tag{5} \end{equation*}
**Step 6:** Perform the uniform distribution by using variance properties. \begin{equation*} V = \left\{ {\begin{array}{c} {\left\{ {{D}_{\left( 1 \right)},{D}_{\left( 2 \right)},{D}_{\left( 3 \right)}\ldots{D}_{\left( n \right)}} \right\},{D}_{ij} < \mu }\\ {\left\{ {{{\tilde{X}}}_1,{{\tilde{X}}}_2,{{\tilde{X}}}_3,\ldots,{{\tilde{X}}}_n} \right\},{D}_{ij} \geq \mu } \end{array}} \right. \tag{6} \end{equation*}
**Step 7:** Generate the denoised image using average of the uniform distribution outcome. \begin{equation*} {Y}_{ij} = mean\left( V \right) \tag{7} \end{equation*}

The image is broken into numerous groups. In one of those combinations, the pixel group is applied to HPWF, and the output pixel is an upgraded version of the original pixel. Various noise sources contaminate each image pixel. Consider the original image ${O}_{ij}$ with its ith row and ${j}{th}$ column pixel values. Here is the generated noisy image ${X}_{ij}$:

\begin{equation*}
{X}_{ij} = {O}_{ij} + {G}_{ij} \tag{1}
\end{equation*}

Convolution with a mask is used to calculate the Gaussian standard deviation (${\sigma }_{GN}$). Here, the convolution operation is performed between Xij and Mask, and generates ${\sigma }_{GN}$. Further, the mask size is chosen based on standard noise threshold and the 3x3 and 5x5 kernels are selected. Then, the mean filtering operation using a local mask with a convolution operation between ${X}_{ij}$ and ${W}_{ij}$ is applied. In addition, the differenced image is estimated by performing a sliding window subtraction operation Moreover, the uniform distribution is performed by using variance properties using distributed outcomes. Finally, a denoised image is generated using the average of the uniform distribution outcomes.

### B. Fusion-Network

**Fig. 2.**
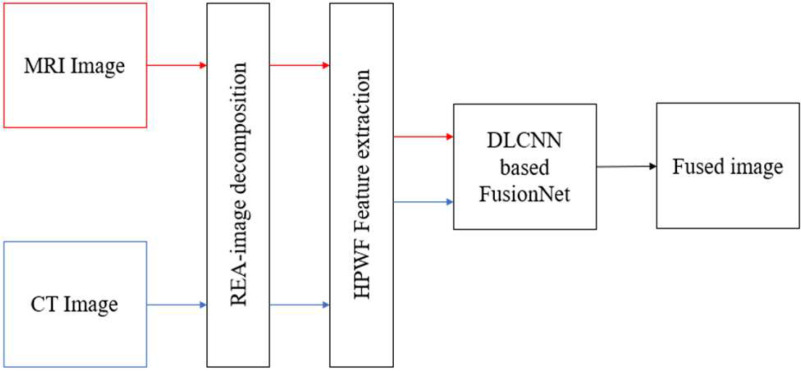
Deep learning convolutional neural network for MRI-CT image fusion.

**Fig. 3.**
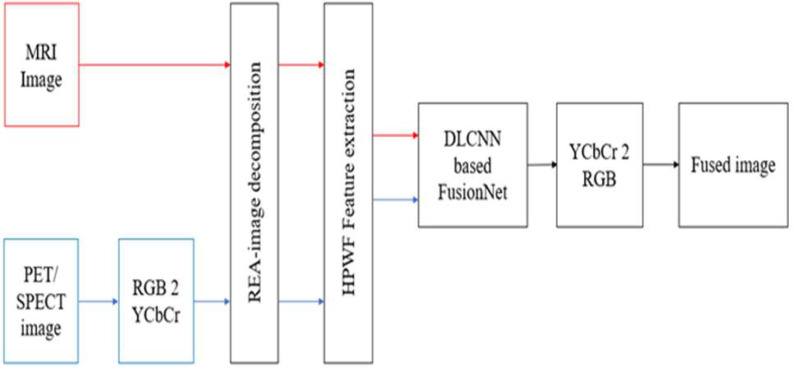
Deep learning convolutional neural network for MRI-PET/SPECT image fusion.

The proposed fusion approach can execute the fusion process among multiple modalities of images, such as combinations of CT and MRI scans, PET and MRI scans, and SPECT and MRI scans, by utilizing two distinct structures to accomplish the operation. Fig. 2 depicts the combination of an MRI and a CT scan, while Fig. 3 depicts the combination of an MRI and a PET/SPECT scan based on a multimodal medical imaging fusion technique.

The REA approach is used for conducting image decomposition. This removes the original slopes and edges of the source features, which enables better slope analysis. if the images are misaligned. In most cases, the slopes of medical features are piecewise smooth, and analysis is used to represent the edges of the image. When aligning these features, it is important to ensure that the edge positions match those on CT data. This demonstrates that the analysis attribute is dynamic and adapts itself depending on the medical features that are referenced to. An active slope analysis feature is the name given to this kind of image. The input feature graphs that were blurry are then removed from the input images that were represented using HPWF in order to generate images that are clear. In addition, the weighted coefficients are derived by the use of the Fusion-Net. After that, in order to arrive at the fused output, the HPWF and REA findings were combined utilizing the weighted mean fusion rule. Table 3 illustrates proposed fusion approach algorithm.

**TABLE III:** Proposed Fusion Approach

**Input:** MRI, CT/SPECT/PET images
**Output:** Fused output
**Step 1:** Apply MRI and CT scans to REA for edge analysis.
**Step 2:** Apply the SPECT and PET based color images to RGB2YCbCr color space format.
**Step 3:** Apply the luminance output of RGB2YCbCr to REA. **Step 4:** REA decomposes the input images and identifies the detailed edges along with its slope.
**Step 5:** HPWF is applied on edges for extracting texture-based features with statistical properties.
**Step 6:** Perform the DLCNN based image feature level fusion using Fusion-Net.
**Step 7:** Preform the YCbCr2RGB color space conversion for MRI-SPECT and MRI-PET based fused outcome using chrominance red (Cr) and chrominance blue (Cb) components.
**Step 8:** Evaluate the various performance metrics from the fused outcome.

### C. Hybrid Fuzzy C-Means Integrated K-Means Segmentation

**Fig. 4.**
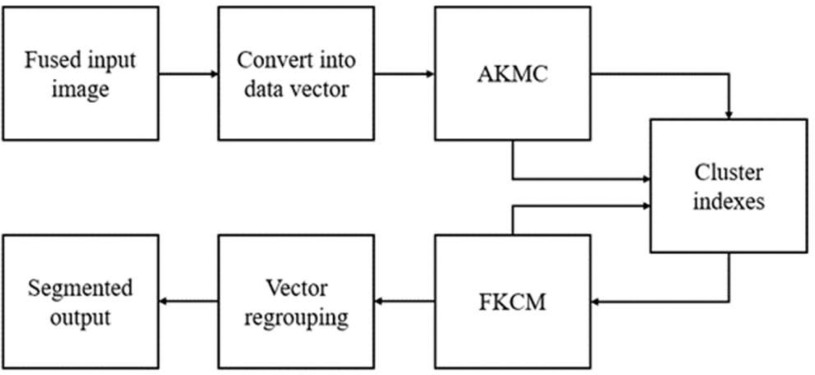
Proposed hybrid fuzzy c-means integrated k-means model.

Brain imaging segmentation is critical in tumor segmentation and analysis. Fig. 4 shows the planned HFCMIK tumor segmentation technique. The restrictions of traditional k-means clustering are solved and maximized by adaptive cluster index localization, which introduces the mean property of cluster centers and similarity matching. This adaptive clustering technique introduces adaptive k-means clustering (AKMC). Each cluster will be used in the fuzzy kernel c-means (FKCM) clustering method for successful brain tumor segmentation.

The HFCMIK algorithm consists of two steps. The AKMC method is used to choose initial centroids in the first phase. Initial centroids are therefore fixed for the whole operation. It reduces the number of iterations needed to group related items. Finally, the AKMC algorithm provides a local optimal by selecting a unique starting centroid. That leads to a global optimal AKMC algorithm solution. The second phase employs the Euclidean distance-based weighted FKCM technique. In both stages, weights associated with each attribute value range in the data set are processed. The upgraded weighted HFCMIK algorithm uses a weighted ranking technique. The weighted ranking method calculates the distance from the origin to each weighted attribute of a data item. The (8) calculates weighted data points.

\begin{equation*}
\mathop \cup \limits_{j = 1}^n = \mathop \sum \limits_{i = 1}^n {W}_i{X}_i \tag{8}
\end{equation*}

where, ${W}_i$ denotes the weightage of ${X}_i$ attribute. For $n$ data points, $n$ numbers of $U$ values are estimated, and the format is given in (9).

\begin{equation*}
\mathop \cup \limits_{j = 1}^n \left( {\begin{array}{c} {{U}_1}\\ {{U}_2}\\ {{U}_n} \end{array}} \right) \tag{9}
\end{equation*}

Then the sorted distances sort the weighted data points (Ui). These data points are then separated into $k$ equal sets, where k is the number of clusters. The initial centroids in each set are the middle points or their mean value. This algorithm's initial centroids lead to cluster members' consistency. Because HFCMIK uses distance measures to choose group members, the proposed weighted ranking algorithm uses the distance formula to pick starting centroids. Thus, the suggested weighted ranking concept is useful for optimizing initial center selection. The unique initial centroid selection and the attribute value-based weights used for processing are two major elements of the first phase of the method. The HFCMIK is a clustered method that breaks an image into clusters. It uses centroids to symbolize its artificially constructed cluster and re-estimates the segmented output. Table 4 presents the HFCMIK algorithm for brain tumor segmentation.

**TABLE IV:** Proposed Hybrid Fuzzy C-Means Integrated K-Means Algorithm

**Input:** Fused image (I)
**Output:** Segmented image (S)
**Step 1:** Convert the input image I into data vectors.
**Step 2:** The initial centroids are identified by the k data points known as cluster centers.
**Step 3:** Label the clusters based on Weighted data centroids as presented using Equation (16).
**Step 4:** perform the weighted sorting operation using Equation (9).
**Step 5:** The distance is computed from ‘U’ to the centroid.
**Step 6:** The closest centroid is assigned as segmented cluster.
**Step 7:** Repeat the process step 3 to step 6 until all clusters covered.
**Step 8:** Combine all the segmented clusters and generate segmented output S.

### D. Hybrid Feature Extraction

It was possible to extract certain features of brain tumor and then utilize those qualities as a basis for categorizing the different lesions. Several essential characteristics have been recovered that have helped in the distinction of brain tumors. These key characteristics include statistical color features, RDWT-based low-level features, and GLCM-based texture features, amongst others. The GLCM technique is a method for assessing textures that takes into consideration the spatial connection between the pixels that make up the brain tumor. The GLCM technique is used to characterize the texture of the brain tumor. This approach works by computing often repeated pixel pairs that have specific values and spectral relationships and then using that information to describe the texture of the brain tumor. Following the completion of the construction of the GLCM, statistical texture characteristics may be extracted from the GLCM using the procedure that was previously outlined. This probability metric establishes the possibility of a certain grey level being found near another grey level. The GLCM characteristics that have been employed include

\begin{align*}
&\text{Contrast} = \mathop \sum \limits_{a,b = 0}^{N - 1} {{\boldsymbol{S}}}_{{\boldsymbol{a,b}}}{\left( {{\boldsymbol{a}} - {\boldsymbol{b}}} \right)}^2 \tag{10}\\
&{\rm{Homogeneity }} = \mathop \sum \limits_{a,b = 0}^{N - 1} \frac{{{S}_{a,b}}}{{1 + {{\left( {a - b} \right)}}^2}} \tag{11}\\
&{\rm{Correlation }} = \mathop \sum \limits_{a,b = 0}^{N - 1} {S}_{a,b}\left[ {\frac{{\left( {a - {\mu }_a} \right)\left( {b - {\mu }_b} \right)}}{{\sqrt {\left( {{\sigma }_a^2} \right)\left( {{\sigma }_b^2} \right)} }}} \right] \tag{12}\\
&\text{Angular}\,\text{Second}\,{\rm{Moment }}\left( {\text{ASM}} \right) = \mathop \sum \limits_{a,b = 0}^{N - 1} S_{a,b}^2\ \tag{13}\\
&{\rm{Energy }} = \sqrt {ASM} \tag{14}
\end{align*}

**Fig. 5.**
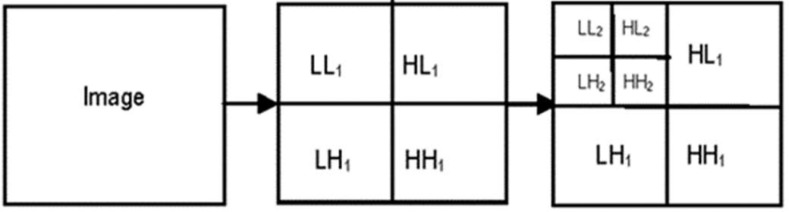
Two-level redundant discrete wavelet transform coefficients.

The next step, which is to extract the low-level characteristics, involves using RDWT with two levels. When RDWT is first applied to the segmented outcome, the output will initially consist of the HH1, HL1, LH1, and LL1 bands in that order. The LL band is then subjected to calculations for its correlation, energy, and entropy properties. After that, RDWT is used once again on the LL output band, which results in the output being LL2, LH2, HL2, and HH2 in that order correspondingly. On the LL2 band, the energy, entropy, and correlation characteristics are computed once more as shown in Fig. 5.

Finally, statistical color characteristics that are based on the standard deviation and mean are retrieved from the feature that has been segmented. They represent it as follows

\begin{align*}
&\text{Mean}\,\left( \mu \right) = \frac{1}{{{N}^2}}\mathop \sum \limits_{i,j = 1}^N I\left( {i,j} \right) \tag{15}\\
&\text{Standard}\,{\rm{ Deviation}}\,\left( \sigma \right) = \sqrt {\frac{{\mathop \sum \nolimits_{i,j = 1}^N {{\left[ {I\left( {i,j} \right) - \mu } \right]}}^2}}{{{N}^2}}} \tag{16}
\end{align*}

After that, all of these features are merged together by means of array concatenation, which produces the output in the form of a hybrid feature matrix.

### E. Deep Learning Based Probabilistic Neural Network Classification

Deep learning models have been more important in the feature extraction and classification process recently. The segmented pictures may be used by the DLPNN models to extract highly correlated detailed spatial, spectral, texture, and color information. The DLPNN models may also recognize the connections between distinct segments of segmented pictures' pixels and extract such connections as features.

**Fig. 6.**
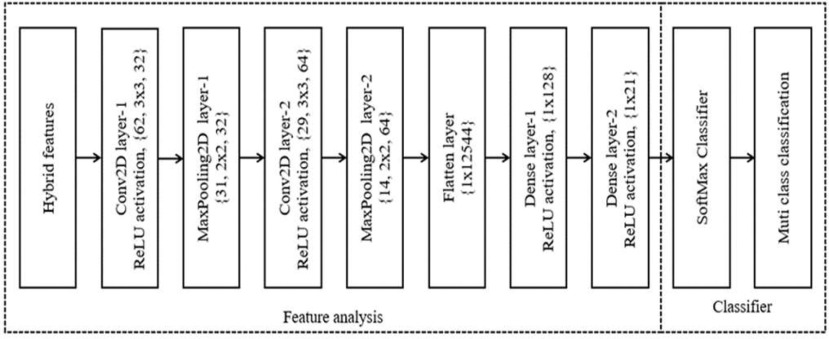
Proposed deep learning based probabilistic neural network classifier.

Finally, the classification operation is carried out by the DLPNN models once they have been trained with these characteristics. In order to create more complex features at lower levels, DLPNN incorporates local characteristics from higher levels of input, making it one of the best possibilities for the classification process. By modifying the weights and kernel sizes in combination with the local connections, the DLPNN may also be made faster. Fig. 6 depicts the DLPNN model for feature extraction and classification. It provides a thorough examination of each layer, including information on the layer's dimension, filter size or kernel size, number of filters, and parameters. A DLPNN model is created by combining all the layers. The DLPNN performs the combined feature extraction and classification of brain malignancies.

## III. Results

This section provides the detailed results analysis and performance comparison with existing approaches using the BraTS-2020 dataset. Furthermore, evaluations of various objective performances and visual objective performances are also presented.

### A. Simulation Environment

This work was simulated using the MatlabR2021a tool with a GPU processor. The models are trained under realistic conditions on the dataset. The simulations are conducted using an NVIDIA Tesla P100 processor equipped with the Windows 10 operating system. The 10-fold cross validation technique is implemented during the training of the proposed model. All the models are trained with MatlabR2021a with 10-fold cross validation through 1000 epochs and a 0.02 learning rate.

### B. Dataset

The performance of the proposed network is evaluated using the BraTS-2020 *dataset* [40]. Multi-modal brain MRI investigations include 369 images for training, 125 images for validation, and 169 images for testing. T1-weighted (T1) contains 80 images, T1ce-weighted (T1ce) contains 80 images, T2-weighted (T2) contains 80 images, and Flair sequences contains 209 images in each study. The annotations for training studies are made available for online assessment and final segmentation competition, but not for validation and test trials.

### C. Performance Evaluation of Proposed Fusion Process

**Fig. 7.**
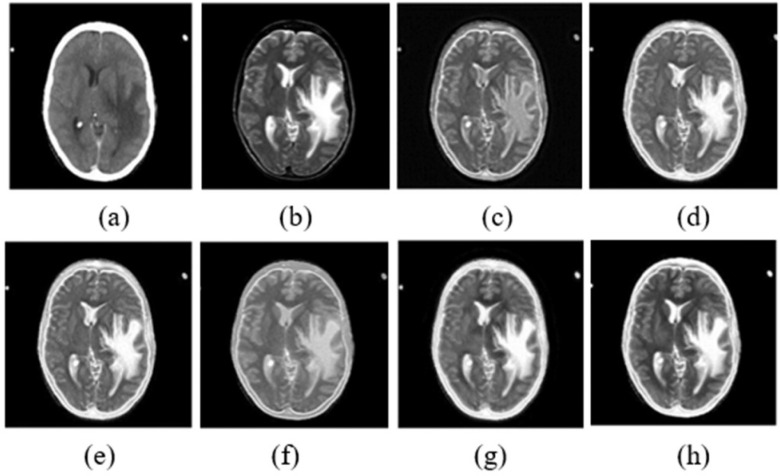
MRI-CT fusion (a) CT input, (b) MRI input, (c) MCA-CS [14], (d) LTEM [15], (e) DB-CNN [17], (f) GF-SDL [18], (g) LDNSD [19], (h) Proposed fused outcome.

**Fig. 8.**
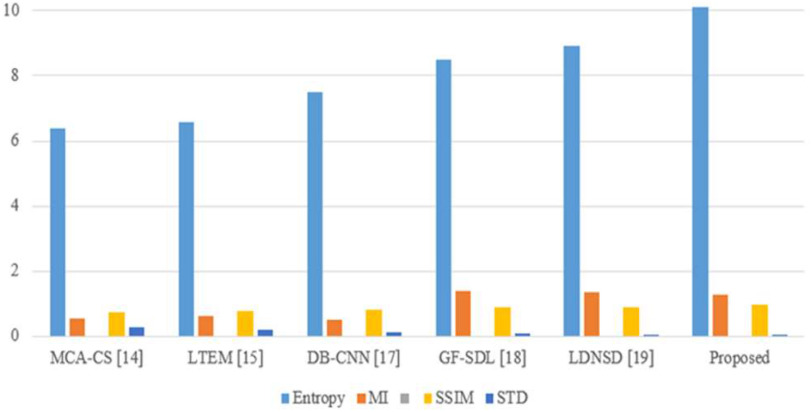
Proposed Graphical representation of MRI-CT image fusion approaches.

The performance is compared by using entropy, structural similarity index metric (SSIM), mutual information (MI), standard deviation (STD), and peak signal to noise ratio (PSNR) metrics. Fig. 7 presents the visual performance comparison of the proposed MRI-CT fusion outcome with conventional approaches like MCA-CS [14], LTEM [15], DB-CNN [17], GF-SDL [18], and LDNSD [19]. Further, the proposed method resulted in higher contrast and brightness with an accurate tumor highlight as compared to other fusion algorithms. Furthermore, Table 5 presents the objective comparison of the proposed method with the existing methods. Finally, for all performance metrics, the proposed approach outperformed conventional approaches in terms of quantitative performance, as shown in Fig. 8.

**TABLE V:** MRI-CT Image Fusion Approaches Performances Comparison

Metric	MCA-CS [14]	LTEM [15]	DB-CNN [17]	GF-SDL [18]	LDNSD [19]	Proposed
**Entropy**	6.374	6.585	7.484	8.485	8.937	**10.2747**
**MI**	0.5647	0.62762	0.489	1.383	1.3536	**1.2784**
**PSNR**	26.438	35.484	41.274	42.447	45.637	**49.337**
**SSIM**	0.736	0.792	0.813	0.893	0.910	**0.972**
**STD**	0.283	0.2012	0.137	0.073	0.0531	**0.0352**

**Fig. 9.**
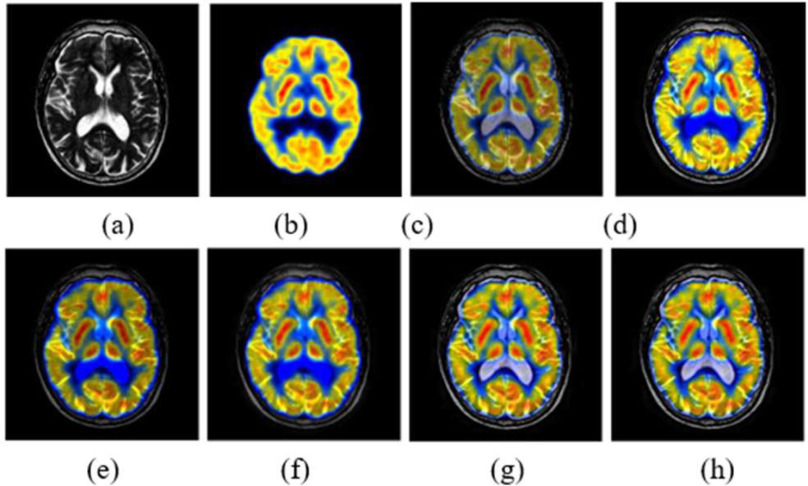
MRI-PET fusion outcomes (a) MR input, (b) PET input (c) MCA-CS [14], (d) LTEM [15], (e) DB-CNN [17], (f) GF-SDL [18], (g) LDNSD [19], (h) Proposed fused outcome.

**Fig. 10.**
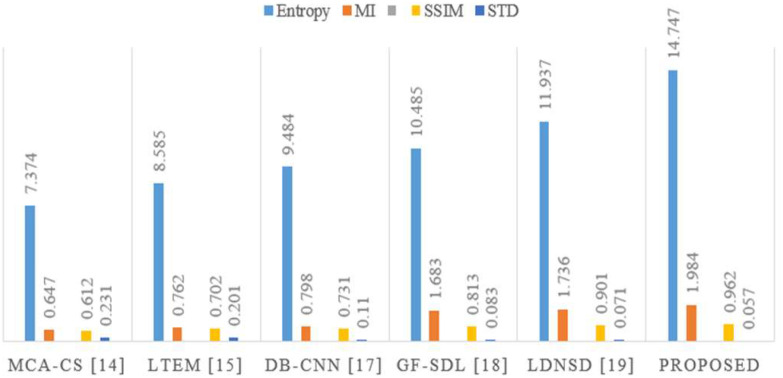
Proposed Graphical representation of MR-PET image fusion approaches.

Fig. 9 compares the proposed MRI-PET fusion result with the visual performance of standard methods such as MCA-CS [14], LTEM [15], DB-CNN [17], GF-SDL [18], and LDNSD [19]. In addition, the suggested approach produced a stronger contrast and brightness, together with an accurate highlight of the tumor, in comparison to the methods that were already in use. In addition, an objective comparison of the suggested approach with the same as other methods that already exist is shown in Table 6. The suggested strategy ended up producing better objective performance than traditional methods for each and every performance parameter, as can be seen in the graphical depiction shown in Fig. 10.

**TABLE VI:** MRI-PET Image Fusion Approaches Performances Comparison

Metric	MCA-CS [14]	LTEM [15]	DB-CNN [17]	GF-SDL [18]	LDNSD [19]	HPWF
**Entropy**	7.374	8.585	9.484	10.485	11.937	**14.747**
**MI**	0.647	0.762	0.798	1.683	1.736	**1.984**
**PSNR**	29.438	37.484	43.274	45.447	47.637	**52.372**
**SSIM**	0.612	0.702	0.731	0.813	0.901	**0.962**
**STD**	0.231	0.201	0.110	0.083	0.071	**0.057**

### D. Performance Evaluation of Proposed Segmentation Approach

**Fig. 11.**
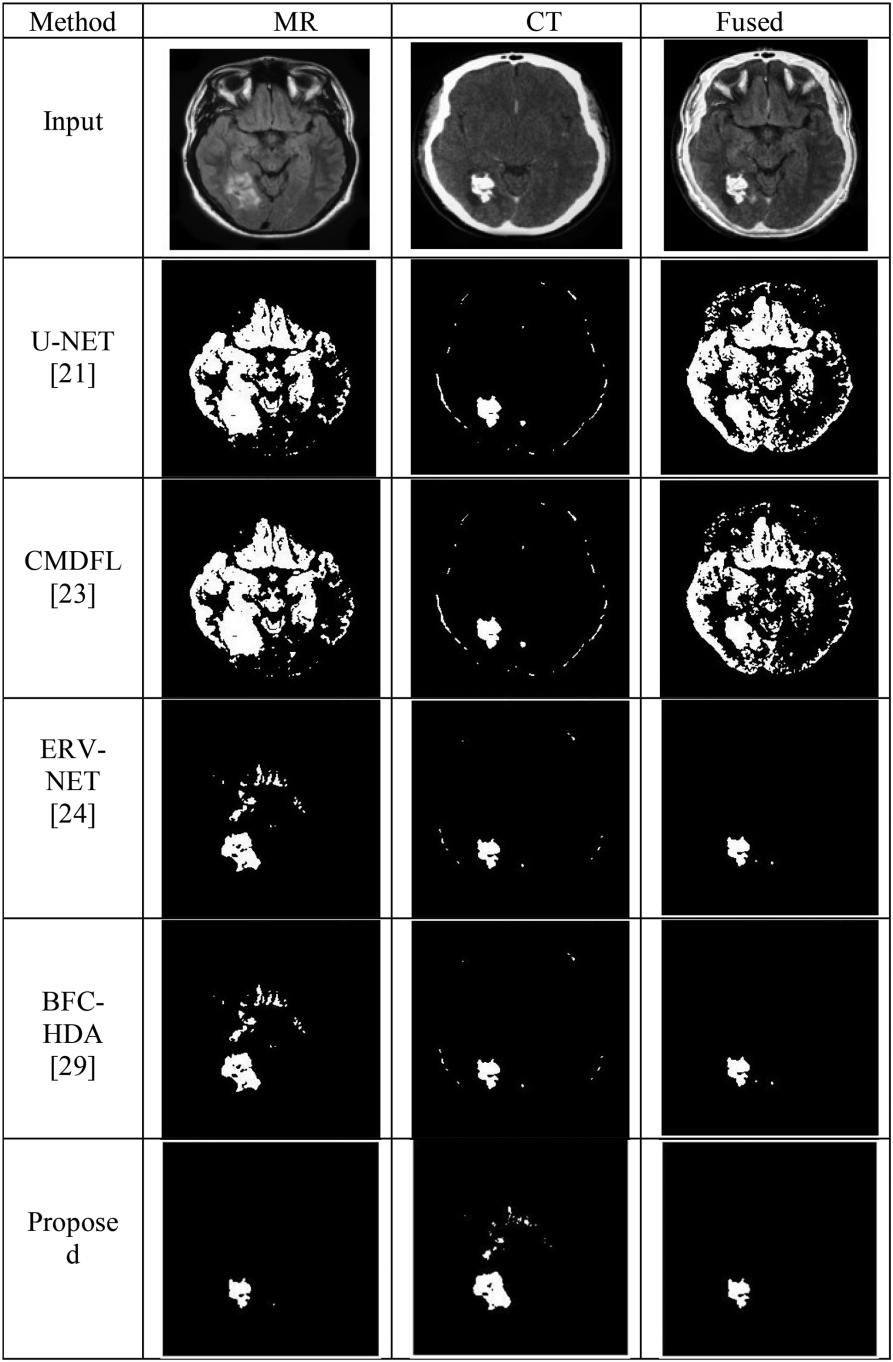
Segmentation performance comparison of MR, CT, and Fused images.

**Fig. 12.**
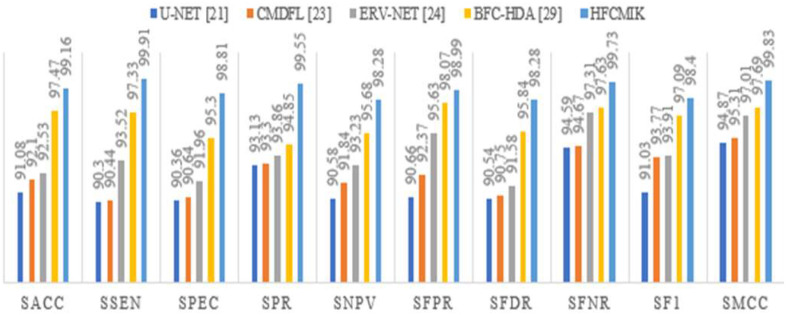
Graphical representation of segmentation performance comparison.

This section provides the detailed simulation analysis of the proposed HFCMIK segmentation approach with conventional approaches. Fig. 12 shows the segmentation performance of various approaches on MR, CT, and fused images. The location of the tumor is not accurately identified using MRI segmentation. Further, CT image segmentation also resulted in poor localization of brain tumors. But the tumor location is perfectly identified by performing the segmentation operation on the MR and CT fused images. Fig. 11 shows the visual segmentation performance of the HFCMIK approach and conventional U-NET [21], K-means, ERV-NET [24], and BFC-HDA [29] approaches. Fig. 11 shows the proposed HFCMIK segmentation approach has resulted in better localization of tumor areas in MR, CT, and fused images as compared to existing algorithms. All conventional approaches are improperly localizing the tumor area.

This article compares the performance of various methods using multiple metrics called segmentation accuracy (SACC), segmentation sensitivity (SSEN), segmentation specificity (SPEC), segmentation precision (SPR), segmentation negative predictive value (SNPV), segmentation false positive rate (SPVR), segmentation false discovery rate (SFDR), segmentation false negative rate (SFNR), segmentation F1-score (SF1), and segmentation Matthew's correlation coefficient (SMCC). Table 7 shows the segmentation performance of the proposed approach with state-of-art approaches like U-NET [21], CMDFL [23], ERV-NET [24], and BFC-HDA [29]. Table 8 shows that the HFCMIK approach outperformed as compared to existing algorithms. The graphical representation of Table 8 is presented in Fig. 12.

**TABLE VII:** Segmentation Performance Comparison of Various Approaches

Method	U-NET [21]	CMDFL [23]	ERV-NET [24]	BFC-HDA [29]	HFCMIK
SACC	91.08	92.1	92.53	97.47	99.16
SSEN	90.3	90.44	93.52	97.33	99.91
SPEC	90.36	90.64	91.96	95.3	98.81
SPR	93.13	93.3	93.86	94.85	99.55
SNPV	90.58	91.84	93.23	95.68	98.28
SFPR	90.66	92.37	95.63	98.07	98.99
SFDR	90.54	90.75	91.58	95.84	98.28
SFNR	94.59	94.67	97.31	97.63	99.73
SF1	91.03	93.77	93.91	97.09	98.4
SMCC	94.87	95.31	97.01	97.69	99.83

**TABLE VIII:** Classification Performance Comparison of Various Approaches

Method	TL-CNN [35]	TL-CNN [37]	FTTL [33]	GAN-VE [39]	DLPNN
CACC	94.84	95.14	96.04	98.06	99.75
CSEN	90.15	95.4	96.62	96.7	99.84
CPEC	91.13	94.26	95.47	97.15	98.18
CPR	92.01	95.06	96.16	97.57	99.47
CNPV	93.87	96.97	97.08	98.38	99.87
CFPR	94.21	96.24	97.1	98.35	99.44
CFDR	90.45	94.91	96.27	96.76	98.96
CFNR	94.83	95.4	96.62	97.95	98.89
CF1	93.41	94.37	95.29	97.92	98.87
CMCC	91.64	93.65	96.29	98.71	99.12

### E. Performance Evaluation of Proposed Classification Approach

This section provides the detailed simulation analysis of the proposed DLPNN classification approach with conventional methods. This article compares the performance of various methods using multiple metrics called classification accuracy (CACC), classification sensitivity (CSEN), classification specificity (CPEC), classification precision (CPR), classification negative predictive value (CNPV), classification false positive rate (CPVR), classification false discovery rate (CFDR), classification false negative rate (CFNR), classification F1-score (CF1), and classification Matthew's correlation coefficient (CMCC).

**Fig. 13.**
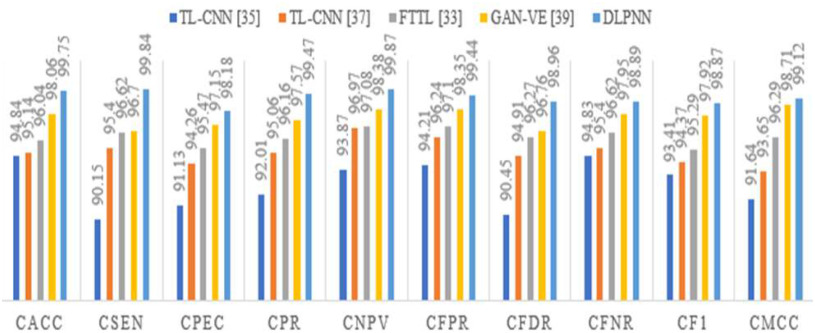
Graphical representation of classification performance.

Table 8 shows the classification performance of the proposed DLPNN approach others methods like TL-CNN [35], TL-CNN [37], FTTL [33], and GAN-VE [39]. Table 8 shows that DLPNN outperformed as compared to existing methods, and the graphical representation of the table is presented in Fig. 13.

## IV. Discussion

As the size of the dataset grows larger, conventional models suffer from high computational complexity. Further, the fusion, segmentation, and classification performance of conventional approaches needs to be enhanced. In the literature, there are multiple deep learning models that have been published. But the complexity of those models is high. So, this work adopted custom deep learning models with unique combinations of layers (convolutional, MaxPooling, ReLU, flatten, SoftMax), number of filters, filter sizes, and stride factor. Finally, all these combinations make the proposed fusion classification model a novel architecture. Furthermore, there is no common method for fusion, segmentation, feature extraction, and classification as of now. Furthermore, in the literature, these combinations of hybrid methods are not published. Therefore, in this work, a novel BTFSC-Net model with the preprocessing, fusion, segmentation, and classification stages has been developed. Initially, HPWF was developed for the removal of various noises from MRI and CT medical images, which also enhanced the contrast, brightness, and color properties. Then, a DLCNN-based fusion network is used to fuse the preprocessed MRI and CT scans with the REA analysis, which improves the region of tumor. Initially, HPWF was developed for the removal of various noises from MRI and CT medical images, which also enhanced the contrast, brightness, and color properties. Then, a DLCNN-based fusion network is used to fuse the preprocessed MRI and CT scans with the REA analysis, which improves the region of tumor. In addition, HFCMIK is used to segment the tumor region from the fused outcome, so an accurate area of brain tumor is detected. Finally, DLPNN is used to classify the benign and malignant tumors from the GLCM and RDWT trained features. The results of the simulation show that the suggested strategy worked better than the other options.

## V. Conclusion

This work implemented a hybrid fusion with segmentation and classification models, which is effectively useful for radiologists to localize the tumor location accurately. This method is also useful in hospitals for computer-aided classification of brain tumors. Initially, this work implemented the HPWF filtering approach for the removal of noise and preprocessing of the source images. In addition, DLCNN-based Fusion-Net was utilized to fuse both source images with multiple modalities. Then, HFCMIK-based advanced segmentation is used to localize the area of the brain tumor. Furthermore, hybrid features were generated from segmented images using GLCM and RDWT approaches. Finally, DLPNN was used to classify benign and malignant tumors by using the trained features. Finally, the simulation-based research findings proved that proposed fusion, segmentation, and classification approaches resulted in superior performance as compared to several conventional methods. Furthermore, the research findings proved that the proposed method can be adopted for real-time applications. Furthermore, this work can be extended with advanced optimization methods for detailed feature extraction.

## VI. Supplementary Materials

### A. Robust Edge Analysis

A REA approach is utilized to accomplish contemporaneous fusion of source medical features throughout the fusion process. The combining of MRI and CT images needs perfect fusion since misalignment is difficult to eradicate. The MRI is initially focalized to increase resolution, allowing for perfect feature fusion. Similarly, lowering the mismatch progressively allows for exact image fusion. These two steps are repeated until convergence is achieved. It also considers the inherent correlation of distinct bands, which was previously overlooked.

A unique active slope approach has been designed for total energy function optimization in these bands. The subproblems are effectively solved using REA with rapid iterations, and the slope extraction was performed via backtracking. Unlike typical variation approaches, REA has just one non-sensitive argument. The initial step in medical image fusion is input medical image fusion. Large medical images need reliable similarity assessment without increasing computing complexity. Thus, REA is used to keep data in space. In addition, any mismatch in the medical imaging would exacerbate the slope analysis. Thus, REA is used to determine similarity.

\begin{equation*}
E = \frac{1}{2}{\left| {\psi R - \bar{M} - \bar{C}} \right|}^2 \tag{17}
\end{equation*}

Here, $E$ is the pixel energy and $R$ represents the edge with slope levels. Since (8) reflects the energy cost of each pixel, a low resolution would result in a large shift, causing image overlapping. To avoid such a superficial solution, the slope extraction approach is used. This may successfully prevent iteratively changing an image in the incorrect direction. This algorithm's goal is to minimize the energy function, as shown in (18). Initially, the problem is fixed as follows for $R$:

\begin{equation*}
E = \xi \left( {{{\left| {\psi R - {{\left( {M - C} \right)}}^{\prime}} \right|}}^2 + {{\left| {\psi R - {{\left( {M + C} \right)}}^{\prime}} \right|}}^2} \right) \tag{18}
\end{equation*}

Finally, $\xi $ is the optimized slope levels. Table 9 illustrates the proposed REA process.

**TABLE IX:** Robust Edge Analysis Algorithm

**Input:** Medical images
**Output:** REA based slope and edges.
**Step 1:** Remove the edge aware noises from medical images using gaussian denoising
**Step 2:** Apply the canny edge detection to identify the different types of shapes, region edges.
**Step 3:** Eliminate the unnecessary edges utilizing average weight distribution-based thresholding.
**Step 4:** Estimate the energy of each threshold region using Equation (17).
**Step 5:** Develop the perfect slope value (ξ) by optimizing the energy aware sub-problems.
**Step 6:** Reduce the unwanted pixels with imperfect energy levels
**Step 7:** Finally, edge aware slopes are generated from non-smoothed regions and smooth textures.

### B. Deep Learning Convolutional Neural Network Based Fusion Network

**Fig. 14.**
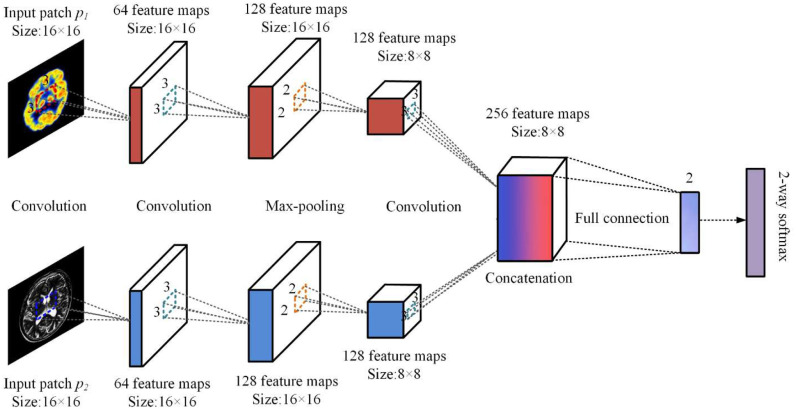
Proposed deep learning convolutional neural network based fusion network.

Deep learning is frequently employed in image segmentation, classification, and fusion applications. Fig. 14 shows a DLCNN-based Fusion-Net architecture for fusing several image modalities depending on features. The Fusion-Net architecture uses convolutional layers. Convolution layers are employed to generate precise features, and the primary purpose of these layers was to perform convolution among the feature patch and kernel-based weight fusion. Convolution layers may be found in neural networks. The convolution layer that is applied to MRI and CT features is represented by (19) and (20).

\begin{align*}
{F}_1 =& \max \left( {0,{W}_1*\left( {{X}_{MR}} \right) + {B}_1} \right) \tag{19}\\
{F}_2 =& \max \left( {0,{W}_1*\left( {{X}_{CT}} \right) + {B}_1} \right) \tag{20}
\end{align*}

Here, W1 is the kernel matrix with its weight values being brought up to date, and B1 is the bias function that is based on the rectifier linear unit (ReLU). In comparison, the convolutional layer kernel only has 3x3 feature maps, whereas the first stage convolutional layer has 64 feature maps that are 16 x 16 in size. Equation (21) is a representation of the ReLU process.

\begin{equation*}
ReLU = \max \left( {0,x} \right) \tag{21}
\end{equation*}

In addition, the features that are generated by the convolutional layers are used as input for the MaxPooling layer, which then meticulously extracts the different types of features. The primary purpose of the MaxPooling layer is to identify the intra-and inter-dependencies that exist among the characteristics of MRI and CT scans.

Utilizing inter and intra dependencies allows for the retrieval of both the effect of CT on MRI as well as the impact of MRI on CT. The operation of the MaxPooling layer is shown by (22) and (23), which may be found below

\begin{align*}
{F}_3 =& \max \left( {0,{W}_2*\left( {{F}_1 + {F}_2} \right) + {B}_2} \right) \tag{22}\\
{F}_4 =& \max \left( {0,{W}_2*\left( {{F}_2 + {F}_1} \right) + {B}_2} \right) \tag{23}
\end{align*}

Here, ${F}_3$ and ${F}_4$ represent the feature maps of the results of the MaxPooling layer for the combined MRI and CT features. In addition, the second stage MaxPooling layers include 128 feature maps that have a size of 16 by 16, and the kernel in the MaxPooling layers has a size of 2 by 2, respectively.

\begin{equation*}
{F}_R = \max \left( {0,{W}_3*\left\{ {{F}_3,{F}_4} \right\} + {B}_3} \right) \tag{24}
\end{equation*}

In this case, ${F}_R$ refers to the fused feature maps that were produced by the concatenation layer, ${W}_3$ denotes the kernel matrix with its updated weight values, and ${B}_3$ refers to the ReLU-based bias function.

**Fig. 15.**
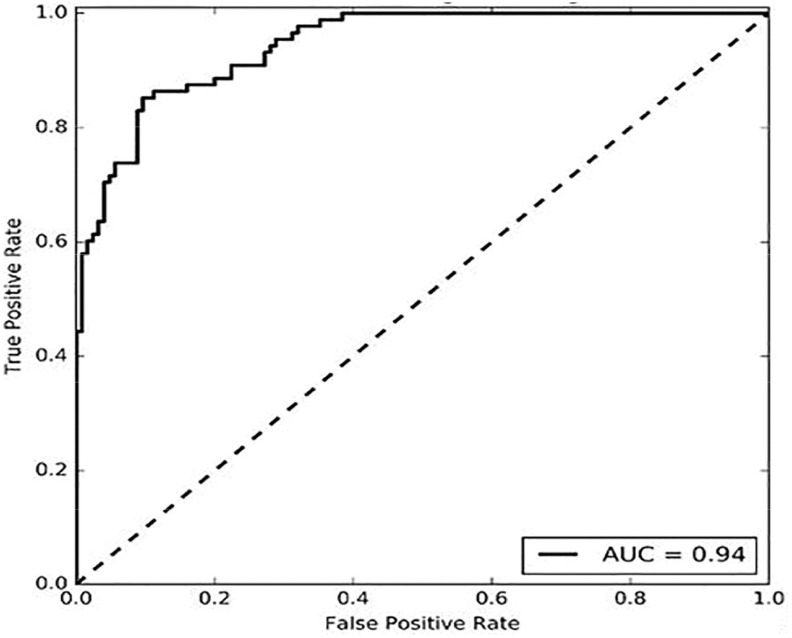
ROC curve with AUC.

Fig. 15 shows the receiver operating characteristic (ROC) curve of the proposed method, which is used to measure the true positive rate and false positive rate of the system. Furthermore, Area Under the ROC Curve (AUC) is also calculated to measure the two-dimensional classification performance of a system. The proposed system achieves an AUC of 0.94.

Table 10 presents the feature values for various methods. Here, the GLCM method calculated the energy and entropy features. Furthermore, the RDWT approach calculated the low-low (LL) and high-high (HH) features. Then, mean and standard deviation (STD) based colour features are calculated. Finally, all the features are combined, which forms the fused feature map. The feature ranking is carried out using a genetic algorithm-based optimal maximization (GAOM) procedure. The individual RDWT, GLCM, and colour features are applied to GAOM, which selects the best features through genetic algorithm-based mutations. Here, the GAOM operation also acts as a feature selection model in the fusion process.

**TABLE X:** Feature Analysis

GLCM		RDWT		Colour		Fused
Energy	Entropy	LL	HH	Mean	STD	Overall
1.3	4.33	56.02	77.34	10.34	9.34	45.35
2.4	5.67	36.93	87.46	11.34	4.56	56.46
3.5	6.56	43.56	56.75	45.35	6.35	67.56
5.6	7.73	54.34	89.56	11.34	7.54	78.45

Table 11 presents the performance comparison of the proposed method without different feature extraction models. The second column contains the proposed model performance with and without GLCM feature extraction. The third column contains the proposed model performance with or without RDWT feature extraction. The fourth column contains the proposed model performance with and without colour feature extraction. The fifth column contains the proposed model performance with and without feature fusion and feature extraction. The Table 11 shows that the proposed method of performance with feature fusion has better performance than other combinations.

**TABLE XI:** Feature Extraction Based Classification Performance

Method	Proposed model without GLCM	Proposed model without RDWT	Proposed model without colour	Proposed model without feature fusion
CACC	96.65	97.88	97.17	96.13
CSEN	97.95	97.49	96.67	96.22
CPEC	97.95	98.68	96.56	97.77
CPR	98.43	96.82	96.78	96.24
CNPV	96.93	96.00	98.06	98.54
CFPR	98.90	96.83	96.05	98.69
CFDR	96.41	97.32	97.25	97.59
CFNR	97.76	98.93	97.32	97.93
CF1	96.57	97.78	98.66	98.92
CMCC	97.35	98.45	98.85	97.43
